# Drone-delivery of defibrillators reduces time to defibrillation in a ski resort: a randomised simulation-based trial

**DOI:** 10.1016/j.resplu.2026.101360

**Published:** 2026-05-13

**Authors:** Michiel J. van Veelen, Tomas Dal Cappello, Giovanni Vinetti, Abraham Mejia-Aguilar, Alexandre Tomasi, Rosmarie Oberhammer, Marika Falla, Giacomo Strapazzon

**Affiliations:** a Institute of Mountain Emergency Medicine, Eurac Research, Bolzano, Italy; b Department of Sport Science, Medical Section, University of Innsbruck, Innsbruck, Austria; c terraXcube, Eurac Research, Bolzano, Italy; d Corpo Nazionale Soccorso Alpino e Speleologico, National Medical School (CNSAS SNaMed), Milano, Italy; e HELI Helicopter Emergency Medical Services South Tyrol, Bolzano, Italy; f Department of Anaesthesia and Intensive Care, Emergency Medicine and Pain Therapy, Hospital of Brunico (SABES-ASDAA), Italy; g Department of Medicine - DIMED, University of Padova, Padova, Italy

**Keywords:** Automated External Defibrillator (AED), Unmanned Aerial Vehicle (UAV), Drone, Basic Life Support (BLS), Emergency Medical Services (EMS)

## Abstract

**Background:**

Out-of-hospital cardiac arrest (OHCA) is the leading cause of sudden, non-traumatic death in ski resorts. Early defibrillation is essential, yet access to automated external defibrillators (AEDs) is often delayed. Drone-based AED delivery has shown promise in urban settings, but its feasibility and performance under winter alpine conditions remain largely unknown.

**Methods:**

In this randomised, simulation-based cross-over trial, three methods of AED delivery were compared: (1) automated drone delivery to bystanders, (2) snowmobile-equipped ski patrol, and (3) simulated helicopter emergency medical service (HEMS) flights. Thirty-six simulations involving 12 bystanders, 2 ski patrollers, and 2 physicians were planned. The simulations took place at distances of up to 2500 m at four locations at 2275 m altitude in a highly visited ski resort in the Alps. The primary outcome was time to defibrillation (TTD). Secondary outcomes included bystanders’ perceived workload, physical effort, and handling difficulty.

**Results:**

Thirty (83%) simulations were completed; six (17%) were cancelled due to severe snowfall. Drone-delivered AEDs achieved significantly shorter TTD compared with snowmobile ski patrol [5.6 (95% CI 4.9–6.3) min vs. 6.7 (95% CI 6.1–7.3) min; *p* = 0.019] and compared with HEMS [15.3 (95% CI 14.6–16.0) min; *p* < 0.001]. Bystanders correctly applied AED pads in all scenarios and reported low median workload (NASA-TLX 7.3/20) and low median handling difficulty and physical effort (VAS 13 and 22 mm on a 100-mm scale).

**Conclusions:**

Automated drone delivery of AEDs in a mountainous ski resort is feasible under winter conditions and could reduce TTD compared with conventional rescue responses.

## Introduction

Out-of-hospital cardiac arrest (OHCA) accounts for the vast majority of sudden, non-traumatic deaths occurring in ski resorts.^1^, ^2^ Early recognition, rapid activation of emergency services, high-quality bystander basic life support (BLS), and early defibrillation form the essential links in the adult Out-of-Hospital Chain of Survival.^3^ Bystander-operated automated external defibrillator (AED) within 3–5 min of collapse—before the arrival of emergency medical services (EMS)—can achieve survival rates of 50–70%^4^ when access to a nearby public access defibrillator (PAD) is effectively facilitated.^5^ The European Resuscitation Council (ERC) BLS guidelines of 2025 state the potential use of drones to speed up the delivery of an AED, especially for areas with longer response times. Real-world studies have demonstrated the feasibility of drone AED delivery, with a time advantage of 1–3 min over ambulances observed in approximately 60% of cases in urban areas.^6^

Epidemiological data from Austrian ski resorts show that approximately 52–53% of deaths are non-traumatic, with 73–99% attributed to cardiac arrest.^1^, ^2^ Compared with OHCAs occurring in surrounding urban areas of the French Alps, arrests on ski slopes involve significantly younger individuals (57 vs. 67 years), are more likely to present with a shockable rhythm, and more frequently occur in the presence of a witness who initiates resuscitation (43% vs. 27%). These characteristics contribute to a higher likelihood of survival, with a reported adjusted odds ratio of 1.96 for 30-day survival among ski-slope cardiac arrests with a favourable neurological outcome.^7^ Prehospital response to OHCA generally relies on helicopter emergency medical services (HEMS) or ski patrol teams,^7^ yet terrain, distance, and weather often result in substantial delays, decreasing the chances of successful defibrillation.^8^

Despite the promising potential of drone-delivered AEDs, no randomised trials have evaluated their feasibility or performance in an operational alpine ski resort under winter conditions. This simulation-based randomised trial is the first to do so, providing a realistic assessment of feasibility under true operational constraints. The aim of this study was to determine whether automated drone delivery of AEDs could be integrated into the emergency response system of a highly visited ski resort supported by snowmobile-equipped ski patrol and regional HEMS. We compared the time to defibrillation (TTD) achieved with drone delivery against that of ski patrol and HEMS, and assessed bystanders’ workload, physical effort, and handling challenges when receiving and using the drone-delivered AED. We hypothesised that drone delivery to bystanders would be feasible and would reduce TTD compared with conventional response modalities. We also evaluated the physical effort, handling challenges, and workload experienced by bystanders receiving and using drone-delivered AEDs.

## Methods

### Study design

**Fig. 1:**
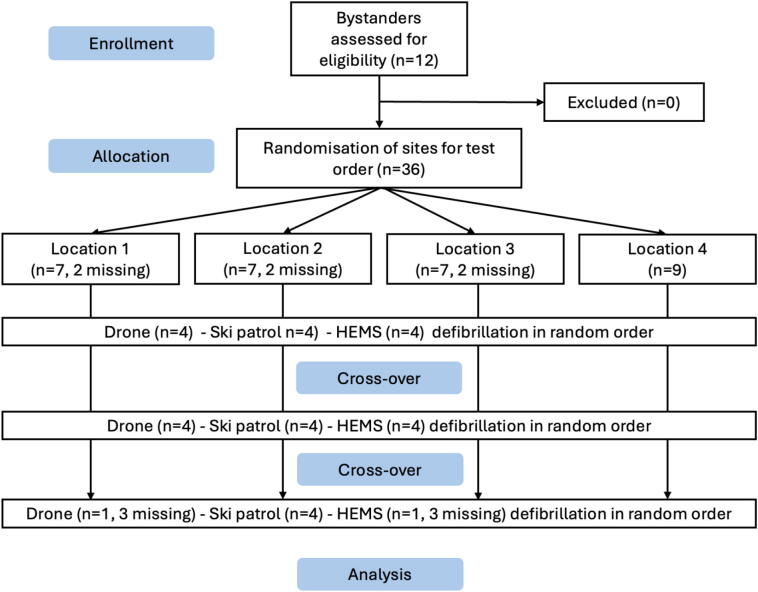
**Study design with enrolment and allocation of participants demonstrating the crossover design of test order (*n* = 36) at the four locations measuring time to defibrillation by drone delivery of the automated external defibrillator (AED) to bystander, ski patrol defibrillation by AED, and helicopter emergency medical services (HEMS) defibrillation**.

This randomised, cross-over, simulation-based trial compared three methods of AED or manual defibrillator delivery and application, and is reported in accordance with CONSORT guidelines (Fig. 1). The study was approved by the Ethics Committee for Clinical Trials and Testing of the Autonomous Province of Bolzano, Italy (protocol 67-2023) and was conducted in accordance with the principles of the Declaration of Helsinki.

### Study setting

**Fig. 2:**
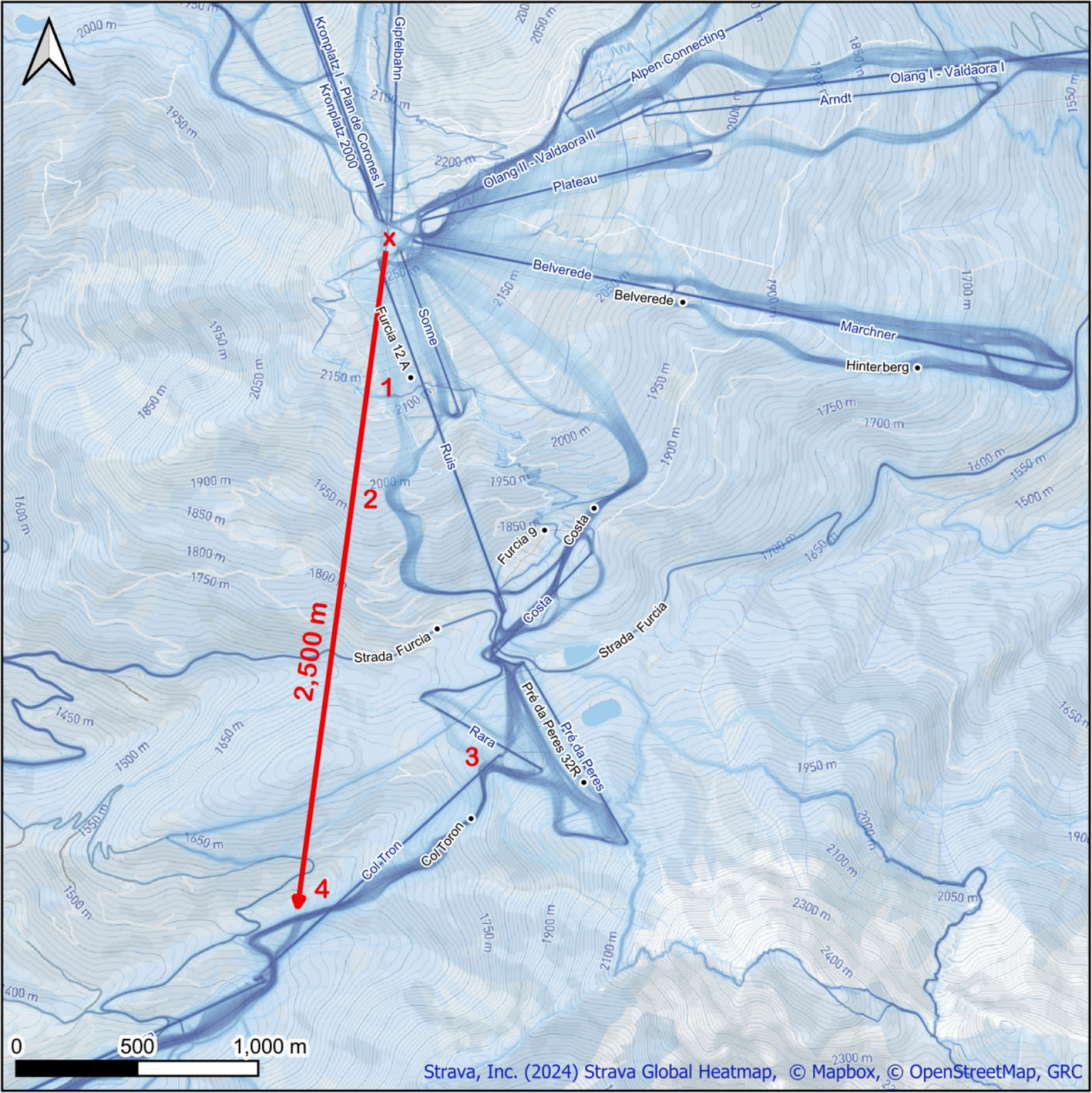
**Study area depicting the study area with curved heat map tracings representing ski slopes (blue), and straight lines represent ski lifts, drone take off point and ski patrol start point (X), and location 1–4 (red) of simulated out-of-hospital cardiac arrest (OHCA). One public road dissects the study area (Strada Furcia). A range of 2500 m flight distance is shown (red arrow). Source Strava, Inc (2024), OpenStreetmap, GRC**. (For interpretation of the references to colour in this figure legend, the reader is referred to the web version of this article.)

The trial took place in the Kronplatz ski resort (South Tyrol, Italy), which receives up to 20,000 visitors per day in high season and comprising 121 km of ski slopes.^11^ The study area lies at 1610–2275 m elevation, approximately 33 km flight distance from the nearest HEMS base, a representative distance compared to other ski areas in the Dolomites. The ski patrol is based on the main summit where it can reach the entire ski resort using snowmobiles. Four locations were randomly selected where cardiac-arrest scenarios were simulated, each between 450 and 2550 m from the drone take-off and ski-patrol start point at 2275 m elevation (Fig. 2). The locations were identified for suitability from a flight permission perspective, based on minimal overpassing of ski area visitors and infrastructure such as roads and ski lifts. The exact altitudes and distances of the locations are given in Table 1. The trial was scheduled independently of weather forecasts to reflect operational conditions, with flights cancelled only when aviation safety limits were exceeded and took place on 9 and 10 April 2024.

**Table 1:** Time to defibrillation (TTD) by means of drone delivery, snowmobile ski patrol and helicopter emergency medical service (HEMS) per location. A hyphen indicates missions not performed because of severe snowfall. SD, standard deviation. For HEMS 33 km is assumed, which is the distance from the nearest base to a central point in the study area.

**Location**	**Altitude (m)**	**Mission**	**Drone**		**Ski patrol**		**HEMS**		**TTD HEMS components**		
			**Distance (km)**	**TTD (min)**	**Distance (km)**	**TTD (min)**	**Distance (km)**	**TTD (min)**	Helicopter flight time*	**Winch needed**†	**TTD from landing**
1	2160	1	0.45	2.8	0.55	5.2	33	16.7	14.0	Yes	2.0
1	2160	2	0.45	6.0	0.55	5.6	33	16.6	14.0	Yes	1.8
1	2160	3	0.45	−	0.55	5.9	33	−	−	Yes	−
2	1990	1	0.95	5.2	1.00	4.5	33	14.3	13.0	No	1.3
2	1990	2	0.95	4.9	1.00	5.2	33	17.2	16.0	No	1.2
2	1990	3	0.95	−	1.00	6.0	33	−	−	No	−
3	1756	1	2.20	5.9	3.10	7.4	33	11.5	10.0	No	1.5
3	1756	2	2.20	5.2	3.10	7.1	33	15.4	14.0	No	1.4
3	1756	3	2.20	−	3.10	7.5	33	−	−	No	−
4	1610	1	2.55	6.9	3.70	9.2	33	14.1	13.0	No	1.1
4	1610	2	2.55	8.4	3.70	8.5	33	18.3	17.0	No	1.3
4	1610	3	2.55	7.1	3.70	8.6	33	13.3	12.0	No	1.3

Mean ± SD	1879 ± 221		1.5 ± 0.9	5.8 ± 1.6	2.1 ± 1.4	6.7 ± 1.6	33 ± 0	15.3 ± 2.1	13.7 ± 2.1		1.4 ± 0.3

* Randomly chosen from a database of 205 historical flight times from the helicopter base in Bressanone to Kronplatz ski resort (time includes activation and take-off).

† Forty-five seconds (i.e., 0.75 min) were added if winch approach was needed.

### Outcomes

The primary outcome was TTD compared between the intervention arm and the two control arms. TTD was calculated as the time elapsed from completion of the emergency call to delivery of the shock (drone intervention and ski patrol control arm). For the HEMS arm, TTD was calculated by adding activation and flight times, modelled from a database of 205 historical missions from the nearest helicopter base in Bressanone, Italy, to the prospectively recorded on-site simulation times (Table 1). Secondary outcomes included assessment of bystanders’ perceived workload, physical effort, and ease of receiving and using the drone-delivered AED.

### Interventions

36 witnessed cardiac-arrest scenarios were simulated at four locations. In twelve scenarios bystanders performed retrieval and application of an AED delivered by drone (intervention arm), and in twelve scenarios two ski patrollers delivered an AED using a snowmobile (first control arm) at four randomly assigned locations in the study area. The drone take-off point and the ski patrol starting point were identical (Fig. 2). In twelve scenarios, two physicians performed a ground approach to the location from either a HEMS landing zone or winch target, depending on the terrain at each location, in random order (second control arm).

The bystanders witnessed a simulated OHCA of a training manikin positioned at a randomly assigned location. They were asked to perform a safety check and make an emergency call, during which they were either directed to wait for a drone delivering an AED at their location or for the arrival of the ski patrol or HEMS. To standardise the AED retrieval process, a study collaborator performed chest compressions during retrieval periods, and bystanders were instructed not to commence BLS until the AED arrived. After the AED application, participants rated their perceived workload using the National Aeronautics and Space Administration Task Load Index (NASA-TLX)^9^ that includes six subscales (0–20) (Supplemental Table S1): Mental Demand, Physical Demand, Temporal Demand, Effort, Frustration (ranging from “Low” to “High”), and Performance (ranging from “Good” to “Poor”). Participants also rated physical effort and handling difficulty using a 100-mm visual-analogue scale (VAS) labelled from ‘low’ to ‘high’ effort or challenge.^10^

The ski patrollers were activated by the dispatch centre directly after the simulated emergency call and began their intervention using a snowmobile.

The physicians performed a HEMS intervention after presumed landing, hovering or winch operation, depending on the terrain. Approach modalities for each location were determined by experienced HEMS providers and the approach times were adjusted accordingly. The prospective time recorded on the ground consisted of the physician reaching the patient while carrying the equipment, attaching the defibrillator pads of the monitor in demo mode and initiating a manual defibrillation.

Before starting the study, all participants were familiarised with the AED, monitor and the pads.

### Participants

Twelve healthy (ASA I) adult volunteers with a valid BLS certification were recruited as bystander to ensure a comparable baseline level of AED operation skills. They were recruited through public notice and selected on a first come first serve basis. Exclusion criteria were age under 18 and ASA greater than I. In the ski patrol arm two rescuers were recruited from the local ski patrol team and for the HEMS arm two licenced physicians were recruited, all using the same inclusion criteria. All participants provided written informed consent.

### Materials

The drone model Q4X (MAVTech, Italy) performed automated flight plans drawn up in Mission Planner (ArduPilot, USA) from the reported coordinates of the distress call. All flights were supervised by a stand-by pilot. The drone carried a polystyrene box attached via a custom bracket and autonomously released it upon reaching the specified GPS location. A self-deploying parachute reduced descent speed to protect the AED. The box contained a Fred Easyport AED trainer (Schiller, Switzerland) with training pads. Once unpacked, the AED operated through standard pre-recorded voice prompts. Simulations used a Resusci Anne QCPR torso manikin (Laerdal, Norway). The HEMS physicians were equipped with a Viper 44 medical backpack (Rock Snake, Austria) and a Corpuls3 monitor-defibrillator (Corpuls, Germany) with a total combined weight of 18.6 kg). Ski patrollers used a Lynx 69 Ranger Alpine snowmobile (Lynx, Finland) and standard patrol clothing and equipment.

### Statistical analysis

A linear regression model was used to analyse the effects of delivery method, location, and their interaction on TTD. Multiple comparisons were adjusted using the Holm-Bonferroni method. Normal distribution was assessed by means of Shapiro-Wilk test and normal Q-Q plots. Normally distributed variables are presented as mean ± standard deviation (SD), and non-normally distributed data as median (range). Estimated means from the regression model are reported with 95% confidence intervals (CIs). All analyses were performed using SPSS version 29 (IBM Corp., USA). A two-sided *p*-value < 0.05 was considered statistically significant.

## Results

Thirty (83%) of the 36 planned simulations were completed (Table 1). Six (17%) simulations—three (8%) drone missions and three (8%) HEMS missions—were cancelled due to severe snowfall (Supplemental Fig. S1). All snowmobile interventions (*n* = 12) were successfully completed despite the weather conditions. All performed drone flights (*n* = 9) achieved successful AED box delivery. AED pads were correctly placed in all scenarios (*n* = 30).

**Fig. 3:**
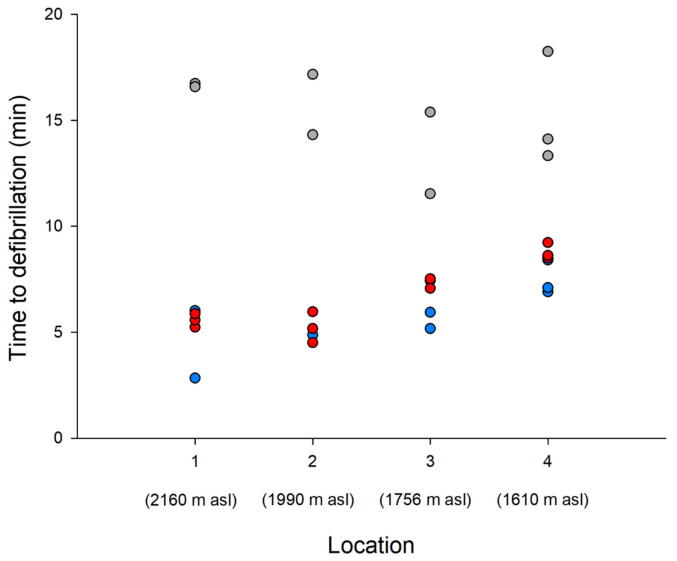
**Relationship between time to defibrillation and location. Every circle represents a mission; blue colour indicates drone delivery; red colour indicates snowmobile ski patrol and grey colour indicates helicopter emergency medical service. asl, above sea level**. (For interpretation of the references to colour in this figure legend, the reader is referred to the web version of this article.)

TTD was normally distributed for each delivery method (*p* = 0.893 for drone delivery, *p* = 0.496 for ski patrol and *p* = 0.885 for HEMS). Drone-delivered AEDs resulted in the shortest TTD, reducing time by 1.1 (95% CI 0.2–2.0) min compared with snowmobile equipped ski patrol [5.6 (95% CI 4.9–6.3) min vs. 6.7 (95% CI 6.1–7.3) min; *p* = 0.019] and by 9.7 (95% CI 8.7–10.7) min compared with HEMS [5.6 (95% CI 4.9–6.3) min vs. 15.3 (95% CI 14.6–16.0) min; *p* < 0.001] (Fig. 3). TTD for the ski patrol was also significantly shorter than for HEMS [6.7 (95% CI 6.1–7.3) min vs. 15.3 (95% CI 14.6–16.0) min; *p* < 0.001]. Both delivery method (*p* < 0.001) and location (*p* < 0.001) had significant effects on time to defibrillation. Their interaction was also significant (*p* < 0.001), indicating that the relative performance of each delivery method varied across the four locations.

Among bystanders, the median physical effort rated on the VAS was 22 mm (range 2–85), and the practical handling challenge was 13 mm (range 3–33) on a 100-mm scale. Overall perceived workload was low, with a NASA-TLX mean score of 7.3 (range 3.2–13.7) on a 20-point scale. No adverse events, operational incidents, or technical malfunctions occurred.

## Discussion

This randomised, simulation-based trial demonstrated that automated drone delivery of AEDs to bystanders in a large alpine ski resort is feasible and can significantly reduce TTD compared with both snowmobile-equipped ski patrol and HEMS. Drone-delivered AEDs enabled bystanders to reach defibrillation approximately one minute faster than ski patrol and nearly ten minutes faster than HEMS, which may represent clinically relevant reductions given the established link between shorter TTD and higher OHCA survival.^12^, ^13^ It has been suggested that reducing the time interval from emergency call to first shock to ≤6 min could be considered a key performance indicator of the chain of survival as every minute of delay to the first shock was associated with significantly lower success rates, and less sustained ROSC during transport.^14^ These findings support the concept that automated drone delivery of AEDs may substantially improve access to early defibrillation, particularly in geographically challenging settings.^3^

To our knowledge, this is the first study to evaluate drone-assisted AED delivery in alpine terrain under winter conditions in an operational ski resort. Our results are consistent with prior research showing that drone-based AED delivery can shorten response times in urban environments.^15^, ^16^, ^17^ A previous simulation trial performed in favourable weather conditions in a highly visited but remote mountainous area also showed significant time gains,^18^ which aligns with the present findings despite more demanding conditions.

As suggested also by the recent ERC BLS Guidelines, drones should be regarded as a complementary component of the Chain of Survival rather than a standalone solution. While early defibrillation is critical, patients also require advanced life support (ALS) and hospital transport. A coordinated response—where drones, ski patrol, and HEMS are activated simultaneously—may therefore provide the greatest overall benefit, ensuring rapid defibrillation followed by definitive medical care and transport. In the present study area, HEMS intervention was associated with delayed initiation of potential defibrillation of more than 15 min, a timeframe known to be linked with poor prognosis.^19^ Consequently, response times to OHCAs occurring in non-urban or rural settings likely benefit from a larger time reduction through drone-delivered AEDs than those in urban environments, particularly in locations with high visitor numbers.^6^ To identify areas with the greatest potential benefit, epidemiological data on cardiac arrest incidence should be combined with geographical information system (GIS) analyses.^20^, ^21^, ^22^, ^23^ Whether drone-delivery of AEDs in ski areas would be cost-effective will depend on real-world TTD reduction and its associated health outcome versus the cost per deployment. However, previous work states that drone delivery in rural areas is more cost-effective than maintaining a network of stationary PADs.^24^

In this study, we selected a location with a high OHCA incidence due to the combination of high visitor numbers engaging in recreational physical activities at moderate to high altitude in cold conditions, and the potential for major time savings given its remoteness. In the Kronplatz ski resort, 22 AED applications were documented in the period between 2013 and 2024, which may possibly be underreported according to ski patrollers (Andreas Testor, White Cross, personal communication, 2024). The current ERC BLS guidelines highlight that no randomised controlled trials have yet investigated drone AED delivery, and ski resorts could therefore represent ideal geographical “hotspots” to validate the real-world clinical effectiveness of drone-delivered AEDs. Full integration of drone deployment into the prehospital care chain, and streamlined flight approval procedures for shared airspace, are essential for timely activation in operational settings.

The Kronplatz ski resort benefits from an exceptionally well-organised ski patrol service operating from a central summit location and neuralgic points in the high season, ensuring rapid coverage of the entire resort. This likely led to the minor time difference observed between drone and ski patrol response. In less centrally structured or topographically complex resorts, with longer access routes, greater time savings can be expected. Drones may offer the most benefit in OHCAs occurring in difficult to reach areas such as off slope locations, where defibrillation times have been reported to be more than double compared with on-slope locations (22 versus 10 min).^7^

The ERC recommends that where drone delivery of AEDs is possible, the dispatch centre should advise bystanders that a drone has been tasked to deliver an AED and provide instructions on retrieving the AED.^3^ Participants in the trial were able to retrieve and operate drone-delivered AEDs with low physical effort, practical handling challenge and overall workload in the present study. These results are comparable to those reported in an earlier trial performed in summer mountainous conditions,^18^ suggesting that snow, cold temperatures, and uneven terrain did not substantially increase mental or physical demand. Severe snowfall prevented deployment on three occasions for both the HEMS and the drone arm, highlighting weather as a limiting factor requiring operational contingency planning. It also highlights the continued essential role of ski patrol services, which remained operational despite severe weather.

This study has several limitations. First, it was simulation-based and may not fully reflect the complex conditions, stressors, and human factors present during real cardiac arrests. Second, HEMS response times were modelled assuming activation from the Bressanone base; in real scenarios, helicopters may already be airborne or positioned elsewhere, potentially altering response intervals. The study dates were predetermined and not altered for weather forecasts, eventually resulting in missing data in the drone and HEMS arms when conditions exceeded operational limits. The small sample size and inclusion of only male participants limit the generalisability of the findings. Furthermore, the inclusion of only BLS-certified bystanders with a preceding familiarisation session may have produced performance levels higher than would be expected in the general population. Drone operations were conducted at reduced flight speeds due to restrictions imposed by the Italian Civil Aviation Authority, as this was the first authorised operation within an active ski resort. The observed TTD reductions were achieved despite submaximal flight performance, indicating potential for even greater gains under optimised regulatory and operational conditions.

## Conclusions

Automated drone delivery of AEDs in a mountainous ski resort environment is feasible and could reduce time to defibrillation compared with conventional rescue responses, except during periods of severe snowfall that restrict aviation operations. Bystanders can safely retrieve and operate drone-delivered AEDs with minimal effort. This technology has the potential to improve early defibrillation access in remote areas where traditional EMS responses are prolonged. Further research in real-world settings is warranted to assess clinical outcomes, operational integration, and scalability across different alpine and rural environments.

## CRediT authorship contribution statement

**Michiel J. van Veelen:** Writing – review & editing, Writing – original draft, Visualization, Validation, Project administration, Methodology, Investigation, Funding acquisition, Formal analysis, Data curation, Conceptualization. **Tomas Dal Cappello:** Writing – review & editing, Visualization, Methodology, Formal analysis, Data curation, Conceptualization. **Giovanni Vinetti:** Writing – review & editing, Project administration, Methodology, Funding acquisition, Formal analysis, Data curation. **Abraham Mejia-Aguilar:** Writing – review & editing, Resources, Project administration, Methodology, Funding acquisition, Data curation, Conceptualization. **Alexandre Tomasi:** Writing – review & editing, Methodology, Data curation. **Rosmarie Oberhammer:** Writing – review & editing, Resources, Methodology, Investigation, Data curation, Conceptualization. **Marika Falla:** Writing – review & editing, Formal analysis, Data curation, Conceptualization. **Giacomo Strapazzon:** Writing – review & editing, Resources, Methodology, Investigation, Funding acquisition, Data curation, Conceptualization.

## Funding

This work was supported by the Fusion Grant tender, promoted by Fondazione Cassa di Risparmio di Bolzano in cooperation with NOI Techpark, Südtiroler Wirtschaftsring and Rete Economia Alto Adige.

## Declaration of competing interest

The authors declare the following financial interests/personal relationships which may be considered as potential competing interests: Michiel J. van Veelen reports financial support was provided by the Fusion Grant tender, promoted by Fondazione Cassa di Risparmio di Bolzano in cooperation with NOI Techpark, Südtiroler Wirtschaftsring and Rete Economia Alto Adige. If there are other authors, they declare that they have no known competing financial interests or personal relationships that could have appeared to influence the work reported in this paper.
