# Supplementary material for: Drone-delivery of defibrillators reduces time to defibrillation in a ski resort: a randomised simulation-based trial
Source: Resusc Plus. 2026 May 13;29:101360. doi: 10.1016/j.resplu.2026.101360 (PMC13218252; doi:10.1016/j.resplu.2026.101360)
Supplement: Supplementary Data 1 [file mmc1.docx]

**
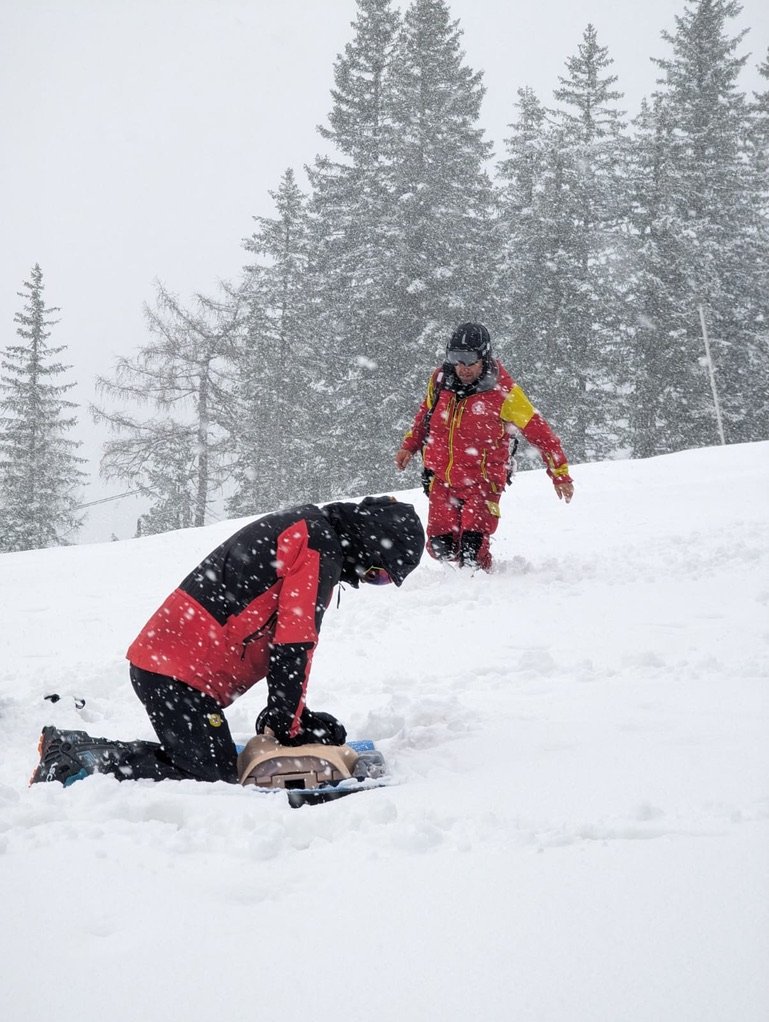
**

**Supplemental Figure S1. Adverse weather conditions**

A ski patroller approaching the site of out-of-hospital cardiac arrest (OHCA) during heavy snowfall. Weather conditions did not permit helicopter or drone flights during part of the trial.
