# Supplementary material for: Drone-delivery of defibrillators reduces time to defibrillation in a ski resort: a randomised simulation-based trial
Source: Resusc Plus. 2026 May 13;29:101360. doi: 10.1016/j.resplu.2026.101360 (PMC13218252; doi:10.1016/j.resplu.2026.101360)
Supplement: Supplementary Data 2 [file mmc2.docx]

**Supplemental table S1. NASA-TLX rating scale definitions**

| **Subscale** | **Description** | **Endpoints** |
| --- | --- | --- |
| Mental Demand | How much mental and perceptual activity was required (e.g., thinking, deciding, calculating, remembering, looking, searching, etc.)? Was the task easy or demanding, simple or complex, exacting, or forgiving? | Low/High |
| Physical Demand | How much physical activity was required (e.g., pushing, pulling, turning, controlling, activating, etc.)? Was the task easy or demanding, slow or brisk, slack or strenuous, restful, or laborious? | Low/High |
| Temporal Demand | How much time pressure did you feel due to the rate or pace at which the tasks or task elements occurred? Was the pace slow and leisurely or rapid and frantic? | Low/High |
| Effort | How hard did you have to work (mentally and physically) to accomplish your level of performance? | Low/High |
| Frustration Level | How insecure, discouraged, irritated, stressed and annoyed versus secure, gratified, content, relaxed, and complacent did you feel during the task? | Low/High |
| Performance | How successful do you think you were in accomplishing the goals of the task set by the experimenter (or yourself)? How satisfied were you with your performance in accomplishing these goals? | Good/Poor |

From: Hart SG, Staveland LE. Development of NASA-TLX (Task Load Index): Results of Empirical and Theoretical Research. In: Hancock PA, Meshkati N, editors. Advances in Psychology. 52. North-Holland; 1988. p. 139-83.
